# Editorial: Therapeutic options in patients with locally advanced non-small cell lung cancer

**DOI:** 10.3389/fsurg.2024.1392081

**Published:** 2024-03-20

**Authors:** Savvas Lampridis, Fabrizio Minervini, Marco Scarci

**Affiliations:** ^1^ Faculty of Medicine, National Heart and Lung Institute, Imperial College London, London, United Kingdom; ^2^ 424 General Military Hospital, Thessaloniki, Greece; ^3^ Division of Thoracic Surgery, Cantonal Hospital Lucerne, Lucerne, Switzerland; ^4^ Department of Cardiothoracic Surgery, Hammersmith Hospital, Imperial College Healthcare NHS Trust, London, United Kingdom

**Keywords:** chemotherapy, immunotherapy, lung cancer, minimally invasive surgery, NSCLC

**Editorial on the Research Topic** Therapeutic options in patients with locally advanced non-small cell lung cancer

## Introduction

Advanced non-small cell lung cancer (NSCLC) represents a significant proportion of lung cancer cases and poses a formidable challenge for clinicians and researchers alike. The management of this patient population requires a multidisciplinary approach that involves various therapeutic modalities. Recent strides in understanding the disease biology and the advent of novel treatment approaches, such as molecular targeted therapies and immunotherapies, have improved patient outcomes. However, the prognosis for patients with advanced NSCLC remains poor, marked by limited long-term survival rates.

With this Research Topic, our aim was to illuminate the latest advancements and clinical insights in the treatment landscape of advanced NSCLC. The contributed articles delve into management strategies and patient outcomes, offering a nuanced perspective on the challenges and prospects in this field. This editorial seeks to distil key findings, recognise limitations, and identify avenues for future research.

## Overview of contributions

Shen et al. conducted an observational study to assess the use of neoadjuvant immunotherapy in patients with resectable NSCLC. Among the 51 patients included, 31 received immunotherapy either as monotherapy or in combination with chemotherapy, while 20 received chemotherapy alone. The findings revealed that immunotherapy led to significantly higher rates of major pathologic response and improved overall survival compared to chemotherapy alone. Importantly, the administration of immunotherapy did not increase surgical complexity or postoperative complication rates.

Zhu et al. investigated the clinical efficacy and safety profile of camrelizumab, a monoclonal antibody against PD-1, as a third- or later-line treatment for advanced NSCLC. The retrospective analysis included data from 257 patients who had failed second-line chemotherapy regimens. Of these, 135 received camrelizumab monotherapy, while 122 received camrelizumab in combination with albumin-bound paclitaxel. The results showed that the combined treatment approach yielded significantly higher objective response and disease control rates, alongside longer median progression-free and overall survival, compared to the group treated solely with camrelizumab. Furthermore, the study identified lines of treatment, metastasis sites, and PD-L1 expression levels as independent prognostic factors for survival.

Pan et al. conducted a comparative analysis of short-term outcomes between robot-assisted thoracoscopic surgery (RATS) and video-assisted thoracoscopic surgery (VATS) for advanced NSCLC following neoadjuvant immunochemotherapy. In their retrospective, single-centre study, 15 patients underwent RATS, while 31 underwent VATS. The findings revealed no significant differences in operative time, resection rates, postoperative complications, or 1-year recurrence-free survival rates between the two surgical approaches. Nonetheless, RATS demonstrated certain advantages, including shorter intensive care unit stays and a more thorough assessment of N1 lymph nodes and nodal stations.

Marziali et al. described their experience with performing right tracheal sleeve pneumonectomy assisted by veno-venous extracorporeal membrane oxygenation (VV-ECMO) for advanced NSCLC in three patients. Employing an anterolateral thoracotomy approach, the surgeons utilized VV-ECMO during the airway reconstruction phase to ensure optimal oxygenation and an unobstructed surgical field. The procedures were devoid of intraoperative complications, and all patients remained recurrence-free at a mean follow-up duration of 27 months.

Zhang et al. performed a literature review focusing on the use of immunotherapy rechallenge (a strategy involving the reintroduction of immunotherapy after discontinuation for any reason) as a treatment option for patients with advanced NSCLC. The authors synthesized research indicating that certain patients may derive benefits from immunotherapy rechallenge, leading to disease control and prolonged survival. Nevertheless, various factors, including patient characteristics, treatment history, efficacy and toxicity profiles of initial immunotherapy, mechanisms of resistance, and the risk of recurrent immune-related adverse events, can significantly influence treatment outcomes.

Wang et al. reported an exceptionally rare case of a giant ovarian metastasis originating from a small primary lung adenocarcinoma in a 43-year-old female patient. This case shows the importance of maintaining diagnostic vigilance regarding synchronous tumours and highlights the necessity of an adaptable treatment approach guided by evolving diagnostic evidence.

## Challenges and future directions

The insights gleaned from the contributions within this Research Topic underscore both progress and ongoing challenges in the management of advanced NSCLC. Despite advancements in therapeutic modalities, several hurdles remain, pointing towards critical areas for future research and clinical practice enhancements.

Personalized treatment strategies represent a cornerstone for improving outcomes in advanced NSCLC. While existing approaches have demonstrated efficacy, tailoring treatments to individual patients through molecular profiling, tumour mutational burden assessment, and immune profiling could further optimize oncologic outcomes while minimizing adverse effects.

Resistance to current therapies poses a significant challenge in the management of advanced NSCLC. As immunotherapy and molecular targeted therapies continue to evolve, understanding and overcoming resistance mechanisms is imperative. Exploring novel combination therapies, alternative dosing schedules, and therapeutic sequencing strategies are avenues ripe for exploration.

The management of treatment toxicities, particularly immune-related adverse events, remains a pressing concern for advanced NSCLC patients receiving immunotherapy. Efforts to enhance early detection, effective management, and prevention of immune-related adverse events without compromising treatment efficacy are paramount for ensuring patient safety and treatment adherence.

Access to novel therapies remains unequal, particularly in resource-constrained settings. Bridging this gap requires concerted efforts to reduce treatment costs, improve infrastructure for molecular testing, and increase awareness among healthcare providers and patients about available treatment options.

The integration of emerging technologies holds promise for further developing the field of advanced NSCLC management. Leveraging technologies such as liquid biopsies, next-generation sequencing, and artificial intelligence, can enhance diagnostic accuracy, predict treatment responses, and monitor disease progression, thereby informing personalized treatment decisions.

Central to these advancements is a commitment to patient-centred care. Prioritizing patient preferences, values, and quality of life in treatment decision-making processes is paramount. Engaging patients in shared decision-making, providing comprehensive supportive care, and addressing psychosocial needs are integral components of holistic care in this cancer patient population.

## Conclusions

This Research Topic has made a useful contribution to our understanding of the therapeutic landscape for advanced NSCLC. The findings emphasize the importance of ongoing innovation and research in this field, with the goal of improving clinical outcomes, prolonging survival, and enhancing the quality of life for patients with this challenging disease.

